# News and Events of the Week

**Published:** 1910-01-15

**Authors:** 


### NEWS AND EVENTS OF THE WEEK.

Mr. George Joseph Cooper, late Liberal M.P. for Bermondsey, who died intestate on October 6, left estate valued for probate at £463. Letters of administration have been granted to his widow.

In the State of Victoria effort is commencing for the stamping out of consumption. Sanatoria are to be built and equipped by the Government, the Municipalities to refund half the money. The first sanatoria will have 100 beds, will cost about £10,000, and about £5,000 a year for upkeep.

Dr. William Roulston, medical officer to Wakefield Workhouse, has been presented with a set of silver candlesticks, one of which bears the following inscription: "Presented to William Roulston, Esq., M.D., M.Ch., M.A.O., by his fellow officers at the Wakefield Workhouse and Infirmary, in recognition of services rendered to them during his 24 years' medical officership."

A Fellow of the Royal Faculty of Physicians and Surgeons of Glasgow has presented the Faculty with a mace, to be used on State occasions.

The Secretary of the Liverpool School of Tropical Medicine received a letter from the President of the Compagnie du Kasai, Brussels, on January 8, enclosing £100 towards the funds in recognition of the work done by the school.

Four Gresham lectures on "Small Pox and Vaccination" will be delivered on Tuesday, January 18, Wednesday, January 19, Thursday, January 20, and Friday, January 21, 1910, by F. M. Sandwith, M.D., Gresham Professor of Physic. At the request of the Gresham Committee the lectures for the present term will be delivered at the City of London School, Victoria Embankment, E.C. (three minutes' walk from Blackfriars Bridge). The lectures are free to the public, and begin each evening at six o'clock.

A feature of the Medical Directory for 1910, issued by Messrs. Churchill, is the list of principal British spas and health resorts, which should prove of value to practitioners for reference purposes. The ordinary lists are as usual up to date and full. There are now 40,558 names of practitioners, as against 36,354 in 1901.

An International Congress on Radiology and Electricity will be held at Brussels in 1910 (September 13 to 15). The work of the Congress will be organised in three sections, the first dealing with Terminology and Radiometry, the second with Physical Science, and the third with Biological Sciences, with a sub-section for Medical Radiology. The following English scientists are members of the General Committee: Sir W. Crookes, Sir W. Huggins, Sir O. Lodge, Sir W. Ramsay, Lord Rayleigh and Professors Rutherford and Schuster. For further details application should be made to Monsieur l'Ingr. Dr. J. Daniel, secrétaire général, 1 Rue de la Prévôté, Bruxelles.

The Women's Medical Association of New York City offers the Mary Putnam Jacobi Fellowship of $800.00 (£160) available for post-graduate study. It is open to any woman graduate in medicine. The fellowship will not be awarded by competitive examination, but upon proof of ability and promise in the chosen line of work. Applications must reach the committee by March 1, 1910, and be accompanied by: (1) testimonials as to good health; (2) letters as to ability and character; (3) educational qualifications; (4) statement of the work the applicant proposes to take up during her fellowship; (5) examples, if any, of her work, in the form of articles or accounts of investigations. Two reports will be expected from the holder, one to be presented about the middle of the research and a detailed one on its completion. All applications should be forwarded to Dr. Emily Lewi, 35 Mount Morris Park, West, New York City, U.S.A.

At an extraordinary meeting of the Fellows of the Faculty of Physicians and Surgeons of Glasgow, a communication from the Secretary for Scotland was read intimating that his Majesty the King has signified his pleasure that the Faculty shall hereafter be known as the Royal Faculty of Physicians and Surgeons of Glasgow. The Chairman said that when they looked back upon the history of their Faculty they found that it started 310 years ago in very humble circumstances indeed. In the last twenty-five years the Faculty had taken part in the examination, along with the sister corporations, and had passed into the profession 4,574 persons. The Faculty was the first, in the northern part of the kingdom at least, if not in Britain, to institute clinical examinations. It was a change in importance second only to the introduction of anatomical study, and it had resulted in a very great and manifest alteration in the education and in the training of students. It did that at a time when there were no hospitals to supply materials for such examinations. They had always felt that in their work with their Edinburgh sister colleges they were at a disadvantage; they were both royal daughters, and although the Glasgow Faculty had the same ancestry, although the Glasgow Faculty was equally brought into being by a royal charter, they (the Glasgow Faculty) had hitherto been a plain Faculty. No doubt it was a unique name, but it was one which the King had been well advised and graciously pleased to honour by the addition of the prefix "Royal."

A petition of Lord Northbrook, President of the Cancer Hospital (Free), and of the trustees and treasurer of the hospital, praying for the grant of a charter of incorporation to the hospital, has been presented to his Majesty in Council; and his Majesty having referred the petition to a committee of the Privy Council, notice is given that all petitions for or against such grant should be sent to the Privy Council Office on or before February 15.

The Vice-Chancellor of London University, Mr. M. J. Muller Hill, gives notice that the election of a member to represent the University in Parliament will occur at the University, South Kensington. All registered graduates, forming the Convocation, if of full age and not legally incapable, should attend. Should a poll be demanded owing to the proposal of more than one candidate, it will take place on Monday 17th, and may be open for five consecutive days, from 8 a.m. to 4 p.m. On the day after the close of the poll, at noon, the Vice-Chancellor will proclaim the result at South Kensington.

Proposed Sanatorium in East Sussex.—The King's interest in the Midhurst Sanatorium has led the local Sussex authorities to consider whether they cannot take a more active part against consumption. The Eastbourne Guardians, with other boards in the eastern division of the county, have formulated a scheme for consideration at a conference at Lewes. It is suggested that the site should be near the South Downs or the sea, and that 100 beds should be provided. The accommodation can be allocated to the various unions in accordance with their assessable value. The area required is from 25 to 50 acres, and the cost of buildings £20,000. The average cost of each patient is estimated at from 25s. to 30s., including the expenses of administration.

A curious cause of death was reported on January 10. An inquest was held at Clapham on Henry Baldry (52), whose death was ascribed to inhaling spirits of salts, used to roughen a slippery granite floor of a school. A house painter said in "laying the floors" he emptied the spirits of salts into a pail, and then used a mop. He had never done it before. The fumes, as he had a respirator on, only made his head ache. Dr. E. H. Drew stated that a post-mortem examination showed death was due to syncope from angina pectoris brought on by indigestion, probably aggravated by the fumes. He believed that this was the first case of the kind on record. The jury returned a verdict according to the medical evidence, and added that there was great negligence on the part of the authorities.

The Seventeenth International Congress of Medicine will be held in London in the year 1913. A General Committee will be formed to carry the meeting to a successful issue and in a manner befitting the reputation of the medical profession in Great Britain and Ireland. Medical men who desire to join this committee should attend the first meeting, which will take place at the house of the Royal Society of Medicine, 20 Hanover Square, London, W., on February 7, 1910, at 5.30 p.m., or communicate with the General Secretaries, Mr. D'Arcy Power and Dr. Clive Riviere. At the special meeting the date of the Congress will be decided, a President, an Honorary General Secretary, or Secretaries, and Honorary Treasurer, and Executive Committee, with power to add to its numbers, will be nominated, and other business transacted.

